# Inebriety

**Published:** 1889-12

**Authors:** Alfred J. H. Crespi

**Affiliations:** Of Wimborne, Dorset


### INEBRIETY.

BY

ALFRED J. H. CRESPI,

*Of Wimborne, Dorset.*

---

THE recent publication of a second edition of Dr. Norman Kerr's classical work on *Inebriety* has aroused much interest in professional circles; for any opinion of that accomplished writer is worthy of, and generally commands, respectful attention. It is significant that a technical and very abstruse work like his should so soon have justified a second edition: it is the best proof of the excellence of the book and the increased attention being paid to the subject; and—may I add?—it also shows that the professional mind is beginning to recognise the true character of inebriety in some cases—that it has many of the features of a disease.

But as Dr. Kerr's work was thoroughly reviewed in this quarterly at the time of its first appearance, a second edition would hardly have called for a second article: the *raison d'être* of my paper is, however, that the Inebriates' Acts, from which so much was naturally expected, expire at the close of the present year, and will have to be renewed. Those Acts have not been so elastic and useful as was hoped; and attempts, it is believed, will be made to introduce fresh clauses, to increase their value. Some of the proposed alterations do not, in my opinion, enhance their value; and in the following article I endeavour to point out a radical change imperatively demanded if real good is to come from the Acts.

Estimates have been frequently attempted as to the number of habitual drunkards of both sexes in the United Kingdom. All place the total high. I shall not frame another, nor formulate a definition of what constitutes habitual intemperance. For my present purpose it is enough that a very large number of persons drink to excess, and injure themselves in health, morals, and pocket; while they are a constant source of anxiety, sorrow, and expense to their relatives. Whether the temperance movement is advancing or not, whether drunkards are more or less numerous, is immaterial to my present purpose, which is the restraint of inebriates, so that their friends may cease to be plagued by them. We have three agencies at work to control drunkards:—the police, who take charge of a large number, and release them after detaining them a few days: retreats under the Inebriates' Acts, which receive a very few victims of this vice, and keep them from mischief, and so far good; but before admission can be obtained the drunkard must voluntarily surrender his liberty, and the magistrate must make him understand the full import of his action: and, in the last place, a certain number of wealthy drunkards are, with infinite pains, and generally at enormous expense, confined in the houses of private friends or in those of medical practitioners. The first system is good and necessary, and, as far as it goes, effectual; but it is only temporary: the second is expensive, and only reaches a very small number. One objection to it is, that the drunkard must surrender his freedom of action and movement; and *that* he will not often do. The third is only suited to the rich: for the medical practitioners who take up this walk of practice only concern themselves with inmates who can pay well; and when it comes to one or two attendants to each resident patient, the expenses range from £300 to £500 a year. Besides, the patient's clothes, books, and miscellaneous requirements have to be provided for, and a grand total of from £500 to £800 a year is reached. This estimate may seem too high; but I quote from the figures of persons conversant with the matter, and from critical inquiries into many cases personally known to me. Busy doctors, preoccupied with professional engagements, will not undertake cases at moderate remuneration, alleging that otherwise they do not pay; so that, to get proper treatment and attention, the charges are enormously in excess of the means of all, save a few rich families; while even then the restraint is not always of real value.

My strictures may seem strong: though I do not mean them as strictures; but can anyone deny that most people engaged in the management of retreats for the intemperate think more of their own profit than of carrying on a great reformatory work? When I first proposed preparing this article, I wrote to many managers and proprietors of such houses, and in no case did I receive any information; indeed, most of the persons addressed did not deign to reply. One or two sent prospectuses; while others favoured me with brief, and not particularly courteous, answers. On writing to one gentleman a second note, he, with charming courtesy, recognising my handwriting, did not open my letter, but forthwith returned it. I would not send an inebriate to an establishment of which I did not know something favourable, and the managers of such homes do not always seem to me the most capable and conscientious persons in the world.

I once saw an officer, whose parents had put him, at his own desire, in the house of a country surgeon in Gloucestershire; indeed, in a house of his own choice. The terms were high—£400 a year; but there was no effectual restraint. The very next day the young man gave his keepers the slip, and was not found for some time. When I was sent for professionally, I found him lying on the floor of a room in a large hotel: he was screaming, throwing himself about frantically, and quite drunk. In another case—that of a profligate officer in South Devon—money was squandered in providing experienced attendants, who were under no real discipline. At last I got the services of a retired corporal-major of the Royal Horse Guards: but no good was done; for the inebriate used to drive to one of the many small towns near, and send his servant to purchase bottles of spirits. In short, the master had the command of money and servants, and was a free agent; and, under the circumstances, attempts to control him were like trying to empty a pond with a sieve.

Perhaps it ought to be made clear that by inebriety is understood habitual excess in any narcotic or narcotico-irritant, like chloral, opium, cocaine, and bromide of potassium. By the way, though perphaps irrelevant, Dr. J. C. Reid, an elderly medical practitioner, now, I believe, deceased, once mentioned in a paper before the British Medical Association that at one time inebriates could be confined in a lunatic asylum for three months. This power seems now taken away. That able practitioner, in the same paper, remarked that, though when he wrote it he was sixty-three, he had only known four drunkards ready of their own free will to go into an inebriate asylum; in other words, a busy and experienced practitioner might not oftener than once in ten years meet with an inebriate ready to sign away his liberty for his own good: that proves that the voluntary co-operation of the sufferer can seldom be counted upon.

We want establishments to receive and detain inebriates on the representation of their friends, and in return for moderate charges. While in the home the patient should be absolutely unable to go out or to obtain stimulants, and his detention should not be a matter over which he had any control: no declaration on his part should be necessary: promptly, cheaply, and certainly, the magistrates should, in suitable cases, have power to commit the inebriate to a proper place for not less than one month nor more than six, not so much for his protection as for that of his friends.

The way must be cleared before approaching the discussion of my plan. An immense amount of sheer nonsense and pseudo-religion is talked on the temperance platform: the drunkard is held up to public sympathy as a poor deluded creature, longing for reformation; and society is often represented as banded against him, tempting him to drink, overcoming his scruples and ridiculing his efforts to avoid temptation. The fact is, that thousands of drunkards are persons of low moral type, with no good resolutions, no pure purpose: they like drink, and will have it: shame they do not feel, and it is absurd to sympathise with them and regard them as suffering angels. Whatever his original position or education, the drunkard is at all times a miserable object, his own deadliest enemy; and as long as we treat inebriety as a failing, we shall do no good. Nor is much gained by looking upon confirmed intemperance as a disease, except in a small percentage of cases: it may modify our view of the sufferer's guilt, but can hardly change the treatment adopted or reconcile his friends to his follies. "You know my failing," said, not long ago, a hopelessly-degraded lady, who for twenty years had been a misery to her family. Her failing, forsooth!—her low, degrading vice, indulged in, in the face of every good influence and of the most excellent surroundings. "Don't let her know that you think she drinks; it would vex her," once said a young lady to me when I was called in to see her mother, a confirmed drunkard, who had got through a bottle of brandy that very morning before breakfast! "Don't scold me; I can't bear it. I am very naughty, I know," I have heard drunkards say.

The drunkard can, or he cannot, control his appetites. If he can, and will not, he is vicious, and should be punished. No doubt the effort required is severe; but in most cases the control, given a sufficiently potent motive, can be exercised; and if the drunkard knew that every act of self-indulgence would be followed, in due course, by a sound flogging, outbreaks would be rare. If the drunkard cannot restrain himself, then he is a lunatic and a menace to society, and he should be treated accordingly. I have seen hundreds of drunkards, and closely followed the career of many for years; and the low tastes, the indifference to the feelings of others, the untruthfulness, and the craving for present self-indulgence at whatever future cost, have dried up my sympathies. Too often the drunkard does not care who pays the penalty of his vices.

"You ought to bring good moral influences to bear," people retort. "Talk to them; reason with them; cheer them; help them to keep their good resolutions." Nonsense! Confirmed drunkards should be locked up, and made to see that they are a menace to society, and must be protected from themselves for the sake of their friends, as well as to give them a better chance.

Then the lawyers interpose: "The liberty of the subject must be respected; it would never do to lock up a man unless with his full consent. Then, what is to be done with his property? Trustees must be appointed; a court of inquiry must sit." In short, if they are listened to, such complicated, costly, and tedious machinery would need to be set in motion, that the Acts would be valueless in the case of countless thousands of inebriates whose circumstances do not admit of vast sums of money being squandered. Is the law generally so merciful? Is the liberty of the subject so religiously guarded? When a man enlists, he soon learns that trifling misconduct entails three months' imprisonment, and that absence without leave is not soon overlooked or expiated. A summons can be taken out against the highest persons in the realm, and they must obey it. It is only the drunkard whose liberty we treat with such scrupulous delicacy. When he is mentioned we feel deep commiseration for him, but not for his friends: he may drink, waste his and their substance, and occasion them great suffering, and we give them no redress, no protection.

"What! punish a man for drinking? Punishment should be reformatory: you should reclaim, not punish." And are the friends to be left out of the reckoning? Are they to suffer a world of misery extending over many years, with no improvement, no reformation? Are parents to pay thousands of pounds, to meet bills wantonly run up, in defiance of strict injunctions to the contrary, by drunken sons?

What we really have is something like this: a large body of working men and women who are drunkards, and who deliberately drink when they can get stimulants; they are too poor to be taken into the present inebriate asylums under the Acts, nor would they consent to enter, and the managers of those institutions do not want them. We have also a large number of men and a fair sprinkling of women of the middle-classes, who are ruining themselves by intemperance. As a rule, these people are irreclaimable, and it is useless to get them to sign the pledge and to attend temperance gatherings. Now and then they conduct themselve fairly well for a time, then comes a relapse. The history of these cases is one of rapidly-accelerating degradation until the end. For all I know, the number of these people may be only 10,000, or it may reach 100,000; probably nearer the latter. Every medical practitioner knows this miserable, degraded class, and has seen scores, perhaps hundreds. I certainly have. "Why," said a drunken officer to me, "should not the poor man have as much beer as will do him good? Why did they call the horse that won the Derby 'Donovan?' Why should public-houses be closed on Sundays?" He thereupon looked at me very sapiently, and propped himself up against a horse to avoid falling. Every night after dinner he is in that condition; his pay, his private means, and the money he can squeeze from an elder brother, go in stimulants; and nothing can be done to restrain him. Another young man sells, when in the humour, everything he can lay his hands on—clothes, books, boots, and college prizes,—and he borrows from anyone, who will trust him, a couple of shillings.

His father pays for a handsome coat, which finds its way to a second-hand dealer's and fetches three shillings; a waistcoat brings in eighteen-pence, and a pair of trousers a couple of shillings; while a costly book commands a shilling. But he would not go into an inebriate asylum, and of course the liberty of the subject must be respected: so that he cannot be forcibly detained except by the police, who occasionally find him drunk, and give him a night in the lock-up; and his friends have to pay, in fines and costs, for the accommodation provided.

The *British Medical Journal*, some months ago, had the following remarkable paragraph, which bears on the subject of this article. I have freely abridged it: "The records of our police-courts, with their enormous mass of frequent offenders or 'repeaters' charged with drunkenness or offences connected therewith, are striking. The remarkable figures published by Dr. J. Francis Sutherland relating to Glasgow, are a lay sermon of ominous significance. We learn that there are 10,000 commitments of women in that city every year. Each of these women is, on the average, convicted three times, and the mean period of imprisonment is seven days. About forty per cent. of these Scottish gaol-birds have had from 11 to 800 previous convictions recorded against them. What does this mean? Simply that, so far from cure or reformation, or even deterrence, these short sentences enable the prisoner to recover from the exhaustion of the last 'drinking-bout,' and send him forth recruited, invigorated, and fit to recommence his vicious indulgence. Such treatment of a pathological condition confirms rather than cures the inebriate habit. The establishment of homes at the public charge, for the scientific care of these police-court *habitués*, would be more economical and effectual."

As I have said above, and as I want to insist upon, we must have a simple, quick, and easy method of dealing with these deplorable cases; and what could be better than the following? Let any person who stands in the relation of parent or child, brother or sister, guardian or trustee to an inebriate—whether the latter is of full age or not—have power to apply at the nearest police-office for a form, in which name, address, occupation, and so on of the applicant and of the inebriate should be fully set forth. A summons should be issued in accordance with these particulars, and be served on the inebriate. When the case came on for hearing, the magistrate should have absolute power to ask for and to obtain information as to the defendant's history, habits, and circumstances. Of course the application should be supported by proper witnesses and reliable evidence. The magistrates would soon eliminate cases in which malice or fraud was the instigating motive; moreover, the defendant could make his defence, and, if possible, clear himself. But, unless I am totally in error, in nineteen cases in twenty, the inebriate would express contrition, seem very much frightened, and promise to amend; he would very rarely, indeed, plead that he was the victim of a conspiracy. Then, according to the circumstances of the case, the defendant should be detained from one to six months, to begin with. When indigent, the expenses should be defrayed out of the rates; when better off, he could have greater privileges, and be charged a moderate—a very moderate—sum. As for places of detention, what could be easier than to provide special departments in gaols or lunatic asylums? At first such special departments need only be added to a few prisons or asylums; after a time, if necessary, to more; and finally special institutions could be built and kept for this class.

What I want to make clear is, that I am not proposing to treat these persons as criminals or lunatics, but as inebriates detained primarily for the protection and relief of their friends: while in the institution they should, of course, have books, papers, and letters, and see their friends, but they should be unable to leave until their sentence had expired. Were such institutions opened, they would soon be crowded, and thousands of families would be relieved of a load of misery.

The fact that the applicants would, in the main, be relations, would prevent outsiders from interfering; but it would also protect the defendants. A father could, for example, set forth that his son had been for six years an inebriate, incurred heavy debts, neglected his occupation, and caused him great anxiety. In many cases the proof of guilt would be overwhelming and unanswerable. Occasional acts of intemperance would be totally different, and would not justify incarceration. Occasionally the charge would not rest on sufficient evidence; sometimes, too, malice might instigate it, but the magistrates would soon eliminate these cases.

As for property, little difficulty could generally arise, for only one person in twenty is said to have any. When, however, the lines had fallen to him in pleasant places, as the inebriate would not be a criminal, he should be at liberty to see letters, documents, and lawyers, and while under restraint could transact any necessary business; so that his friends would not have the unrestricted control of his property, though they might use some of his things. If the wish to appropriate his property was the principal motive of the action, the defendant would be the very person of all others most likely to know; and a clever lawyer would be retained, who would not neglect to make the most of the information given him by his client. But the property difficulty would not often arise; for in the vast majority of cases the very ground of the application would be the extravagant and bad habits of the inebriate, and the injury he was doing his connections, and detention would be demanded for the protection of his friends. At most, it could only be a question of the use of horses, books, and houses for a very short time, and even that would in great measure be got over by the inebriate being able to give directions as to their management. If he refused to conform to the law or to the necessities of the case, and, as might occasionally happen, would not make proper arrangements, his connections might apply, at his expense, for the temporary management of his affairs to be entrusted to a relative, lawyer, or friend; though as soon as the magistrates were satisfied as to his habits his detention should commence. This question of the management of property is that one, however, on which the lawyers will do their best to wreck the scheme: no one sent to prison for a few weeks for some trifling lapse from the right path is allowed to plead for acquittal on the ground that he has a horse or a house; and yet I fear the custody of a little property, in one case in twenty, will be held to be a serious matter, and will ruin the proposed reform, unless the detention of inebriates is approached on sound common-sense principles. When the property is considerable, and the inebriate has been confined several times, provision might be made in the Acts for the appointment of trustees with almost absolute control.

One class, and a very large one too, I have not dealt with—that of confirmed low-class inebriates, whose friends might not always trouble to move, or perhaps there might not be any friends to take the initiative. Here I think the local police authorities should take the first step and instead of sending a person to prison 200 times, as frequently happens, they could apply in due form to the magistrates for an order to commit the inebriate to an institution for six months; the expense, when necessary, being defrayed out of the rates. A friend of mine has sent a woman to gaol thirty-six times for being drunk and disorderly. I contend that the law urgently needs alteration, and that it is simply disgraceful to see the same wretched inebriate in the dock time after time. Something much more effectual and dignified should be attempted.

A great deal will be made of the disgrace to the family, and we shall be told that forcible detention in the inebriate institution would be insufferable. Does anyone suppose that a man's habits are a secret to his friends, servants, employers, and neighbours? Many rich families delude themselves with the belief that the habits of a scapegrace son or an abandoned daughter are a profound secret, while the decorous servants, who are alone supposed to be behind the scenes, have long ago told all the other servants in the neighbourhood the whole tale; and in this way half the inhabitants of a small town may be in the secret, and follow its development curiously.

The need for such institutions is urgent. The present Inebriates' Acts could go on; private medical practitioners, with relays of highly-paid attendants, could be resorted to in proper cases; and the police might still look after the occasional riotous drunkard; but we require something more effectual to reach the overwhelming mass of inebriates, who cannot afford to pay a medical man for very imperfect supervision vast—I do not say exorbitant—sums; for the treatment of such cases in private houses is harassing and expensive, and must be liberally paid for to make it worth while embarking upon it.

Were some of our temperance associations to turn their attention to this matter, and promote the passing of an Act that would meet the requirements of the case, they would do a great and useful work, and perhaps, in the long run, promote temperance more effectually than by a crusade carried on chiefly among people who have already signed the pledge and have no temptation to break it. Temperance meetings do not reach inebriates. Much must be left to the discretion of the magistrates; for an attempt to frame an Act so comprehensive that it would cover the whole ground, and deal with all possible difficulties and complications, would lead to its being so cumbrous and complicated that, though it would find ample occupation for the lawyers, it would not protect the public. Moreover, as the magistrates would have full power to dismiss the application on the ground of insufficient evidence, unsuitability, or malice, the liberty of the subject would be effectually protected. Lastly, in cases of possible fraud or malice, the penalties attaching to perjury would be incurred by the applicants, and conspiracy need not often be feared.

Such an extension of the Acts would give them the necessary elasticity and completeness, and make them a boon to the nation, instead of, as at present, a delusion.

